# The Dynamics of Shannon Entropy in Analyzing Climate Variability for Modeling Temperature and Precipitation Uncertainty in Poland

**DOI:** 10.3390/e27040398

**Published:** 2025-04-08

**Authors:** Bernard Twaróg

**Affiliations:** Department of Geoengineering and Water Management, Faculty of Environmental and Energy Engineering, Cracow University of Technology, 31-155 Cracow, Poland; bernard.twarog@pk.edu.pl; Tel.: +48-602129566

**Keywords:** Shannon entropy, bootstrap method, NOAA data, monthly precipitation, mean temperature, climate dynamics, entropy attractor, copula function

## Abstract

The aim of this study is to quantitatively analyze the long-term climate variability in Poland during the period 1901–2010, using Shannon entropy as a measure of uncertainty and complexity within the atmospheric system. The analysis is based on the premise that variations in temperature and precipitation reflect the dynamic nature of the climate, understood as a nonlinear system sensitive to fluctuations. This study focuses on monthly distributions of temperature and precipitation, modeled using the bivariate Clayton copula function. A normal marginal distribution was adopted for temperature and a gamma distribution for precipitation, both validated using the Anderson–Darling test. To improve estimation accuracy, a bootstrap resampling technique and numerical integration were applied to calculate Shannon entropy at each of the 396 grid points, with a spatial resolution of 0.25° × 0.25°. The results indicate a significant increase in Shannon entropy during the summer months, particularly in July (+0.203 bits) and January (+0.221 bits), compared to the baseline period (1901–1971), suggesting a growing unpredictability of the climate. The most pronounced trend changes were identified in the years 1985–1996 (as indicated by the Pettitt test), while seasonal trends were confirmed using the Mann–Kendall test. A spatial analysis of entropy at the levels of administrative regions and catchments revealed notable regional disparities—entropy peaked in January in the West Pomeranian Voivodeship (4.919 bits) and reached its minimum in April in Greater Poland (3.753 bits). Additionally, this study examined the relationship between Shannon entropy and global climatic indicators, including the Land–Ocean Temperature Index (NASA GISTEMP) and the ENSO index (NINO3.4). Statistically significant positive correlations were observed between entropy and global temperature anomalies during both winter (ρ = 0.826) and summer (ρ = 0.650), indicating potential linkages between local climate variability and global warming trends. To explore the direction of this relationship, a Granger causality test was conducted, which did not reveal statistically significant causality between NINO3.4 and Shannon entropy (p > 0.05 for all lags tested), suggesting that the observed relationships are likely co-varying rather than causal in the Granger sense. Further phase–space analysis (with a delay of τ = 3 months) allowed for the identification of attractors characteristic of chaotic systems. The entropy trajectories revealed transitions from equilibrium states (average entropy: 4.124–4.138 bits) to highly unstable states (up to 4.768 bits), confirming an increase in the complexity of the climate system. Shannon entropy thus proves to be a valuable tool for monitoring local climatic instability and may contribute to improved risk modeling of droughts and floods in the context of climate change in Poland.

## 1. Introduction

Entropy, a fundamental concept in physics, plays a crucial role in the analysis of climate systems. In its thermodynamic interpretation, entropy describes the degree of disorder and energy dissipation, whereas in its informational form (Shannon entropy), it quantifies uncertainty and complexity within data systems, such as time series of temperature and precipitation. Recent studies indicate that thermodynamic and informational entropy are closely related, enabling new approaches to analyzing climate variability.

Thermodynamic entropy is central to the functioning of Earth’s climatic “heat engine” [1,2]. This system is not in thermodynamic equilibrium, as temperature and pressure gradients drive atmospheric and oceanic circulation, resulting in the transfer of heat and moisture [3,4,5]. The operation of this system leads to the production of entropy through various processes, such as dissipation of kinetic energy in the atmosphere (e.g., due to wind friction and aerodynamic drag); phase changes like condensation of water vapor, sublimation of ices, or melting of snow; and diffusion of heat and moisture, including vertical heat transport between atmospheric layers [4].

Shannon’s information entropy enables a quantitative assessment of uncertainty and randomness in climate data [6,7]. Its analysis of temperature and precipitation time series allows for the detection of changes in variability, which is particularly important in the context of forecasting extreme weather events [7]. There is a strong relationship between thermodynamic and informational entropy—an increase in thermodynamic entropy may be linked to more complex climate processes, leading to greater informational unpredictability of the data. Consequently, analyzing long-term trends in informational entropy becomes a powerful tool for identifying structural shifts in climate and assessing potential future directions of its evolution [8,9,10].

Climate data—especially those related to temperature and precipitation—form the foundation of research on climate change [11]. They are used across various sectors of the economy and environmental policy, including the following:

- Agriculture—for drought forecasting, optimizing irrigation systems, and managing agricultural production [12];
- Water management—for predicting water resource availability, managing retention, and implementing flood protection strategies [13];
- Energy—for assessing water availability for cooling power plants and forecasting energy demand [14];
- Spatial planning—in evaluating flood risks and protecting infrastructure [12];
- Medicine and public health—for anticipating the effects of heatwaves and humidity changes on human health [15].

These datasets support the analysis of trends, detection of anomalies, and identification of extreme climate events, such as severe storms, heatwaves, prolonged droughts, or floods [16]. Variations in temperature and precipitation directly affect Earth’s energy balance and, thus, influence entropy production within the climate system [3,17].

Calculating informational entropy based on temperature and precipitation distributions enables a deeper understanding of structural changes in the climate system [18,19]. This is particularly important since traditional statistical methods often fail to capture shifts in variability and randomness within climate data [6,20]. Monitoring entropy over long time series allows the identification of regions particularly vulnerable to climate change—for example, areas at risk of more frequent droughts or heavy rainfall. An upward trend in entropy indicates growing instability and unpredictability in climate behavior and can serve as a basis for long-term forecasting of extreme events. Such analyses are especially valuable for projecting future patterns of temperature and precipitation, which is crucial for water resource management, urban climate adaptation, meteorological risk assessment (e.g., heatwaves, severe storms, thermal anomalies), and the development of adaptation strategies in sectors such as agriculture, forestry, and critical infrastructure [21,22].

Poland lies within a temperate transitional climate zone and is experiencing significant climatic changes that influence the distribution of temperature and precipitation. Between 1951 and 2021, the average temperature increased by approximately 0.28 °C per decade, with an even more pronounced rise in winter months—reaching 0.36 °C/decade [23]. The number of hot days is increasing, particularly in the northern regions, leading to a higher risk of droughts [24,25,26]. Meanwhile, the number of frost days is decreasing, especially in northeastern Poland [27]. Precipitation changes are less uniform, but forecasts suggest an increase in the number of days with extreme rainfall, especially in the eastern parts of the country [28,29]. The variability of temperature and precipitation in Poland exhibits characteristics of chaotic systems [30,31,32]. Extremes in these parameters can be attributed to the interaction of various circulation systems, involving air masses with differing thermal and humidity properties [26,33]. Additionally, the increasing frequency of weather anomalies—such as violent storms or prolonged droughts—contributes to the chaotic nature of climate change [34,35]. This growing variability further underscores the relevance of informational entropy as a tool for detecting and monitoring such changes [36]. Among numerous previous studies on climate variability in Poland, the works of Falarz, Niedźwiedź, and Opała-Owczarek [37,38] primarily focused on statistical analyses of temperature and precipitation trends. Particularly influential were the studies conducted by Kundzewicz, Robson, and Miętus [23,39], as well as those by Cebulska and Twardosz [24,25], Wałęga and Młyński [26,33], Łupikasza and Małarzewski [40], Ustrnul, Wypych, and Czekierda [41]. Although the application of informational entropy has been mentioned in works by da Silva and Hao [18,30,42], it has rarely been combined with a copula-based approach.

The limitations of classical statistical methods and the absence of frameworks capable of integrating complex interdependencies between climate variables formed the basis for the present study. The choice of the Clayton copula to model asymmetric dependencies between precipitation and temperature was inspired by its applications in hydrological and financial analyses, such as those by Nelsen and Hao [18,43,44].

These studies demonstrated that modeling extremes and dependency structures requires moving beyond linear correlation. Additionally, the use of phase space and entropy trajectories draws inspiration from research on chaotic systems—particularly those by Lorenz [45,46] and Silva and Bhattacharya [47,48]. A thorough review of these methodological approaches enabled the development of a framework tailored to Polish conditions, incorporating both temporal and spatial dimensions. As a result, this study represents a synthesis and advancement of several research perspectives, offering a novel tool for assessing climate variability.

## 2. Data Preparation for Analysis

The analysis presented in this article is based on daily gridded climate data derived from publicly available products provided by the National Oceanic and Atmospheric Administration (NOAA) [49,50,51]. This study covers an area of approximately 1225 km by 670 km, encompassing the territory of Poland (latitude: 49° to 55° N, longitude: 14° to 25° E) [52]. A uniform spatial resolution of 0.25° × 0.25° was adopted for the analysis [53]. The dataset used includes monthly mean air temperature and monthly total precipitation [52].

In this study, NOAA data were used directly in their original resolution (0.25° × 0.25°). For each grid cell, separate time series of monthly temperature and precipitation were extracted and served as the basis for the entropy-related analyses conducted over the period 1901–2010 [49,54].

To ensure high data quality, consistency, and comparability across both time and space, this study relied on climate datasets from the NOAA repository, which applies standardized quality control protocols. This dataset was selected for its homogeneity—both precipitation and temperature records were developed at the same spatial resolution, by the same institution, and using unified interpolation algorithms. NOAA data are routinely validated for anomalies, gaps, and inconsistencies using statistical procedures and are cross-checked against ground-based observations and satellite measurements [50,51,52,54]. As a result, a reliable and methodologically consistent dataset was obtained, providing a solid foundation for long-term analyses of Shannon entropy trends.

## 3. Methodology

The Earth’s climate system is driven by the continuous flow of energy resulting from temperature and pressure gradients. A growing body of scientific evidence indicates that human activity significantly influences the formation and evolution of these gradients, thereby intensifying climate change processes [4,30]. As a result, we observe shorter periods of intense precipitation and prolonged phases of high temperatures combined with low rainfall. The variability of extreme events—such as floods and droughts—has become increasingly evident, and this intensification can be attributed to both natural climatic processes and anthropogenic influences [26].

In the present study, monthly precipitation totals and mean monthly temperatures were analyzed, with particular emphasis placed on their seasonal patterns and variability at the monthly scale. This approach allowed for an expansion of the data scope, an increase in observation frequency, and a more objective and critical assessment of the cyclic patterns of temperature and precipitation [55,56].

This methodological choice offers several important advantages:

- First, the use of monthly values ensures greater statistical stability, appropriate frequency for assessing periodicity, and improved detection of seasonal cycles. Statistical stability is especially critical in long-term studies, where excessive data variability may lead to erroneous interpretations and suboptimal decision-making [57].
- Second, analyzing average values increases the number of usable observations, making the results more representative of long-term trends [58].
- Furthermore, this approach better reflects the underlying climate reality, as it focuses on typical conditions rather than isolated extremes, which may distort the broader picture of climate variability. Mean values enhance statistical robustness and capture a wider range of observations, thereby increasing the accuracy of long-term trend assessment. Such an approach is essential for informed decision-making and effective climate adaptation planning [34,56].

To model the joint distribution of temperature and precipitation, a bivariate Clayton copula function was employed [18,59]. For each 0.25° × 0.25° grid cell, long-term time series of precipitation and temperature were constructed, and through optimization involving different marginal distributions, the parameters of the marginals and the copula were estimated. Due to the limited sample size, a bootstrap resampling technique was applied to assess the variability distributions of Shannon entropy, enhancing the reliability of the results obtained [60,61].

## 4. Bootstrap Resampling Technique

In this study, the bootstrap resampling technique was applied to estimate the distribution parameters of seasonal precipitation and temperature values. This method is favored not only for its computational efficiency but also for its conceptual simplicity, which allows for the generation of bootstrap replications without relying on assumptions regarding the underlying distribution [61]. It operates solely based on information derived from the sample data.

Sequences of monthly precipitation and temperature values were constructed. However, the number of elements in these sequences was insufficient to allow for a reliable estimation of Shannon entropy within a 95% confidence interval. Therefore, a total of 1000 bootstrap resamples were drawn from the original 70-element sequences [36]. To assess the trends in Shannon entropy, it was assumed that the 70-element sequences were constructed recursively. For the estimation of monthly Shannon entropy, the analysis was based on the values generated through the bootstrap sampling approach:

(1) $${X^{P}}_{i}={P^{month}}_{1900+i},..,\;{P^{month}}_{1970+i}\;,\;i=1,\ldots ,40,\;{X^{T}}_{i}={T^{month}}_{1900+i},..,\;{T^{month}}_{1970+i},\;i=1,\ldots ,40$$

In this way, forty sequences of seventy elements each were arbitrarily constructed. These sequences served as the basis for 1000 bootstrap resamples. A total of 40 datasets were generated, each subjected to 1000 bootstrap iterations. For each resample, the Shannon entropy was calculated within a 95% confidence interval. This procedure was applied to every grid cell under analysis. The described methodology was implemented across all 396 cells of the study area. The analytical code was developed using MATLAB software (version R2021a).

## 5. Fitting the Normal and Gamma Distribution

The modeling of Shannon entropy variability in this study incorporates values for phenomena such as precipitation and temperature [62]. A commonly applied approach in entropy-based modeling involves extracting sequences of observations from consistent time periods—such as monthly cumulative precipitation values. This method is similarly applied to average monthly temperatures, under the assumption that the datasets are independent and identically distributed. These datasets are then fitted to appropriate probability distribution models.

For monthly temperature data, the normal distribution proved to be the best fit, as confirmed by Anderson–Darling tests (ADT) [8,62]. In the case of monthly precipitation sums, the gamma distribution was found to be most appropriate. This probabilistic characteristic of precipitation over the Polish territory was also confirmed using the ADT. Parameters for both the normal and gamma distributions were estimated separately for each dataset using the Maximum Likelihood Estimation (MLE) method [63,64,65]. This methodology has been widely studied and is considered a “user-friendly” and robust approach in statistical analysis.

Normal distribution [65]:

(2) $${f(x,\sigma ,\;\mu )}_{S}=e^{\frac{-{\left(x-\mu \right)}^{2}}{2\sigma^{2}}}$$

where x, σ, μ are, respectively, temperature as a random variable, standard deviation, and mean value.

Gamma distribution [65]:

(3) $${f(P/a,b)}_{S}=\frac{1}{b^{a}\Gamma (a)}y^{a-1}e^{\frac{-y}{b}}$$

where y,a,b, Γ(a) are, respectively, precipitation as a random variable, distribution parameters, and Gamma function.

## 6. Fitting the Copula Clayton Function

Copula functions are statistical tools used for multivariate modeling of probability distributions of random variables. Initially applied in the field of economics, copulas enable the construction of a joint multivariate distribution by combining marginal one-dimensional probability distributions of individual variables. The concept itself is not new—it was introduced in statistical theory over 80 years ago by Hoeffding (1940, 1941) and Widder (1941) [66,67,68].

Thanks to the ability to merge marginal distributions and the increasing computational power available, the theory of copula functions has gained growing recognition and is now widely applied across various fields of engineering and applied sciences. In many statistical or probabilistic decision-making problems, a fundamental question arises: is there a joint multivariate distribution consistent with known marginal distributions? Copula theory provides a mathematical framework to address this question (Nelsen, 1999; Joe, 1997; Drouet Mari and Kotz, 2001) [43].

Typical examples from the Archimedean family include the Gumbel–Hougaard copula (both single- and two-parameter forms), the Frank copula, the Clayton copula, and the Plackett copula. Other commonly used copulas come from the elliptical family, such as the Gaussian copula and the t-copula [44,68,69,70].

The Clayton copula was selected in this study due to its ability to model strong dependencies in the lower tail of the distribution. This property is particularly important for analyzing phenomena such as droughts, which involve extremely low precipitation accompanied by high temperatures. Alternative copulas such as Gumbel or Frank are better suited to modeling upper-tail dependencies or moderate correlations, making them less appropriate in cases of the asymmetric dependence observed in this research. The Gaussian copula assumes symmetric dependence, which does not adequately reflect the often nonlinear relationship between precipitation and temperature in the Polish climate. Therefore, the Clayton copula offers the best fit for the data and is justified both theoretically and empirically.

A commonly used measure in such analyses is Kendall’s tau coefficient (often denoted as τ_Kendall) [43]. Unlike Pearson’s correlation coefficient, which measures linear relationships and assumes normally distributed data, Kendall’s tau assesses monotonic dependence between variables and performs well in the presence of nonlinear relationships. It is also directly linked to copula functions—Kendall’s tau can be expressed in terms of copula parameters, facilitating the fitting of an appropriate copula model to the data. Moreover, it demonstrates robustness to outliers, as it is based on data ranks rather than absolute values, making it less sensitive to extreme observations than Pearson’s correlation [71]. See Table 1.

In this study, the Clayton copula was adopted to construct the joint probability distribution based on the normal and gamma marginal distributions [72]. The copula parameter θ was optimized by fitting the theoretical cumulative distribution function (CDF) to the empirical CDF using the minimum squared error (MSE) criterion [73,74]. The theoretical CDF was derived from the normal and gamma marginal distributions, whose parameters were estimated using the Maximum Likelihood Estimation (MLE) method. The results of the parameter optimization for θ yielded the monthly values presented in Table 2.

The average values of the copula parameter θ indicate the strongest dependence between precipitation and temperature during the winter months (January: 0.361; November: 0.295; December: 0.293), suggesting that these variables are more closely correlated in this period due to the presence of more stable atmospheric patterns (Table 2). The lowest θ values occur in summer, particularly in July (0.025) and September (0.016), indicating a weaker relationship, which may result from greater variability in weather conditions, such as local convective storms, irregular precipitation, or heatwaves. The observed decreasing trend from January to September, followed by an increase in autumn, points to seasonal variability in the strength of dependence, which should be taken into account when modeling extreme weather events and their impact on the climate system.

## 7. Shannon Entropy

Although originally rooted in information theory, Shannon entropy is widely used in environmental and climate data analysis as a nonparametric measure of uncertainty, disorder, and variability of probability distributions. In this study, it was applied to describe monthly distributions of temperature and precipitation without assuming any specific distributional shape (e.g., normality). Rather than being used to analyze time series dynamics in the classical sense (such as with ARIMA or GARCH models), entropy serves here as a tool to characterize the degree of unpredictability of climate variables within discrete time windows. For each monthly sample, an empirical distribution is constructed, from which entropy is calculated. The resulting time series of entropy values is then analyzed with respect to trends, change points, and spatial differentiation.

In the climate science literature, information entropy has been successfully applied in studies of seasonal and spatial climate variability, as well as for detecting regime shifts in atmospheric patterns. This study emphasizes an empirical assessment of entropy evolution over time and space as an indicator of local climate instability—without the need for comparison to an “ideal” reference distribution, which is often difficult or impossible to define in the context of complex, nonlinear climate processes.

While more advanced information–theoretic measures such as the Kullback–Leibler divergence are available, their application requires specifying a fixed reference distribution [75]. In the context of highly variable meteorological data, this can lead to interpretive ambiguities. Therefore, this study adopts a self-contained approach based on empirical entropy, enabling the monitoring of disorder in weather data without imposing additional model-based assumptions.

From an informational perspective, a rising trend in Shannon entropy signifies greater randomness and complexity in the distribution of climate variables. This directly translates into decreased forecastability of weather conditions and potentially greater vulnerability of the system to extreme events. As such, using entropy as an indicator in climate analysis aligns well with the growing interest in nonparametric measures of variability, which support the investigation of irregularity and risk in an evolving climate system.

Shannon entropy quantifies the uncertainty associated with predicting the value of a random variable [20,76,77]. It is computed based on the probability distribution of the data, and its accuracy depends heavily on the precision with which this distribution is estimated. Incorrectly estimated distributions can result in misleading entropy values, thereby affecting the reliability of conclusions drawn about the system under investigation.

The Shannon entropy for a two-dimensional continuous random variable (*x*, *y*) with a joint probability density function *f*(*x*, *y*) is defined as follows [71,78,79]:

(6) $$H_{S}\left(X,Y\right)=-\int_{R^{2}}f(x,y)\log_{2}f\left(x,y\right)dxdy$$

Here, $f\left(x,y\right)$ is the joint PDF of the bivariate distribution derived from the copula and marginals. Marginal PDFs: the Normal and Gamma distributions, $f_{X}\left(x\right)$ and $f_{Y}\left(y\right)$. Copula density c(u,v), which is derived from the Gaussian copula [77].

The formula for the joint PDF becomes

(7) $$f\left(x,y\right)=c(F\left(x\right),G(y))f_{X}\left(x\right)f_{Y}\left(y\right)$$

Use the definition

(8) $$H_{S}\left(X,Y\right)=E(\log_{2}f\left(X,Y\right))$$

to estimate the entropy as the negative mean of the log joint PDF.

In discussions surrounding the units of Shannon entropy for continuous distributions, results are typically expressed in information units such as nats (derived from natural logarithms) or bits (using base-2 logarithms) [36]. Despite its widespread application, it is important to recognize the limitations and potential pitfalls associated with the use of Shannon entropy. Several noteworthy constraints include the following:

- Sensitivity to measurement scale: Entropy calculations are affected by the scale of measurement. The units used can significantly impact the computed entropy, necessitating precise definitions and appropriate scaling.
- Assumption of uniform distribution: In meteorological data, assuming a uniform distribution of all outcomes can be problematic—especially since variables like precipitation are naturally bounded. This can lead to underestimation of entropy.
- Neglect of inter-variable correlation: Ignoring correlations between variables such as temperature and precipitation can result in oversimplified models that fail to capture the true complexity of the system.
- Data discretization: The process of binning or categorizing data influences entropy calculations. The chosen discretization method must align with the nature of the data to ensure accurate entropy estimation.

This study focuses on the computation of Shannon entropy for monthly precipitation sums and average temperatures, using numerical integration techniques. The sequences generated from these calculations formed the basis for further analysis of entropy variability under different climatic conditions.

Anticipating criticism regarding the limitations of Shannon entropy, the methodological framework was carefully designed to address these known issues. This included the following:

- Standardization of measurement units;
- Careful selection of marginal distribution parameters;
- Application of a uniform discretization methodology across all datasets.

To validate the empirical distributions of temperature and precipitation against their theoretical models, the Anderson–Darling (AD) goodness-of-fit test was applied. These steps ensured that the dataset met the rigorous statistical standards necessary for robust analysis of climatic phenomena.

## 8. Statistical Tests Used

To assess entropy trends for both precipitation and temperature, bootstrap resampling techniques were employed to generate sequences for the calculation of Shannon entropy. For each realization, the parameters of the marginal distributions and the joint distribution—constructed using the Clayton copula function—were estimated separately. The characteristics and patterns of these trends were evaluated using the Mann–Kendall test (MKT) at a 5% significance level [80,81]. In addition, change-points in entropy trends were identified using Pettitt’s test (PCPT), also at the 5% significance level. Where a change-point was confirmed, the new trend for the subsequent subsequence was re-assessed using the MK test [82,83,84]. The suitability of marginal distributions describing temperature and precipitation values for each analyzed sequence was verified using the Anderson–Darling Test (ADT), also conducted at the 5% significance level.

The Mann–Kendall (MK) test is commonly used to detect monotonic trends in time series data and has been widely applied in climate change research [80]. The magnitude of the trend is estimated using the Sen’s slope estimator, a non-parametric method proposed by Sen [85] and extended by Hirsch [29,86]. In this study, the MK test was used to evaluate trends in Shannon entropy for both temperature and precipitation.

Several methods can be used to identify change-points in time series [82,84]. Pettitt’s test compares the ranks of two subsets of data divided at a potential change-point to determine whether a statistically significant shift has occurred. This test is appropriate for data that do not follow a normal distribution, as it does not rely on normality assumptions. The result is determined by comparing the test statistic to the critical value at the desired significance level, providing a decision on whether to reject the null hypothesis of no abrupt change. PCPT has been widely used in the detection of change-points in climatic and hydrological time series [83].

In the present study, Pettitt’s test was applied to detect abrupt shifts in the time series of Shannon entropy, calculated from monthly precipitation sums and average monthly temperatures. For sequences where a statistically significant change-point was detected, the trend analysis was applied to each subsequence separately; otherwise, the trend test was applied to the entire sequence [36].

## 9. Analysis of Shannon’s Entropy Trend Variation

The study employed a joint distribution model based on a copula function, describing the combined variability of monthly mean temperature (assumed to follow a normal distribution) and monthly precipitation sums (assumed to follow a gamma distribution) [4,18,87]. The Clayton copula was selected for entropy analysis due to its effectiveness in capturing strong lower-tail dependence, which is particularly relevant for assessing extreme events such as concurrent low precipitation and high temperature—typical of drought conditions. In contrast, the Gumbel copula better models upper-tail dependencies (e.g., extreme precipitation during high temperatures), which is more appropriate for analyses focused on changes in mean values [44,70,88]. Although the t-copula provided a comparable fit in some cases, the Clayton copula was better suited for representing dependencies in the lower tail, which was essential for the drought-focused context of this study [12,13]. On the other hand, the Frank copula models moderate dependence well, but lacks the ability to capture specific tail characteristics, making it less effective in describing extreme weather conditions [12].

The calculated Shannon entropy pertains to the joint distribution of temperature and precipitation. This means that entropy does not allow for a direct separation of the sources of variability in temperature and precipitation individually, but rather describes the overall uncertainty and disorder in their joint probability structure. Interpretation of entropy trends therefore requires an approach that considers both structural changes in the climate system and the dynamics of interdependence between temperature and precipitation. An increase in entropy may reflect rising unpredictability in their relationship—for instance, a weakening or destabilization of previously stable correlations between the two variables. This could point to shifts in climatic mechanisms, such as alterations in atmospheric circulation patterns, changing seasonal cycles, or the impact of global warming on precipitation intensity across different temperature ranges.

Assessing Shannon entropy trends for the joint distribution of precipitation and temperature provides an important tool in climate research [7,89]. Historical data analysis enables the identification of directional changes in the unpredictability of the climate system and offers deeper insight into the dynamics of its regional evolution [90]. Statistical methods such as entropy trend analysis enhance the accuracy of climate forecasting and may inform adaptive planning strategies in the face of changing climate conditions.

## 10. Results of the Analyses and Discussion

**Figure 1:**
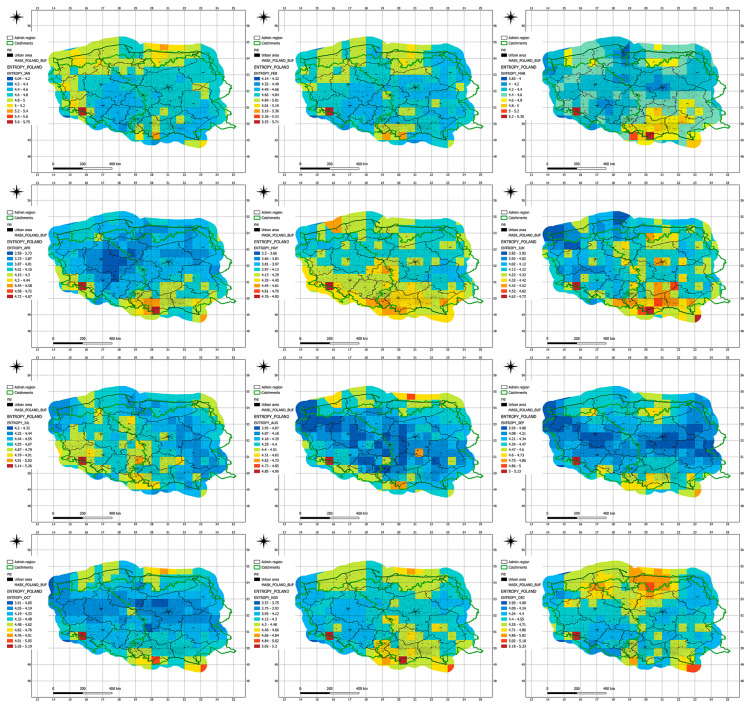
Seasonal Shannon entropy values calculated for the most recent period (1941–2010).

To clearly assess entropy trends for both precipitation and temperature, a bootstrap resampling method was applied to generate Shannon entropy sequences. Shannon entropy was calculated based on the joint probability distribution (Figure 1). The estimation of parameters for the marginal distributions representing the variability of temperature and precipitation was performed using the Maximum Likelihood Estimation (MLE) method. The significance of these trend patterns was verified using the Mann–Kendall Test (MKT) at the 5% significance level. Additionally, the Pettitt Change-Point Test (PCPT) was applied at the same significance level to detect any shifts in entropy trends. When a change point was confirmed at the 5% level, a new trend form for the subsequent data subset was defined using the MKT. The adequacy of the marginal distributions for each sequence of values was assessed using the Anderson–Darling Test (ADT), also at the 5% level of significance.

The results are presented graphically, providing a clearer and more precise visualization of the evolving entropy values and their associated trends.

### 10.1. Analysis of Shannon Entropy Values in Second-Order River Basins

**Figure 2:**
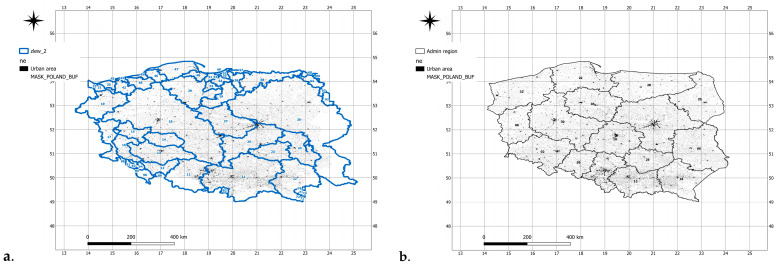
Location of river basins (**a**) and public administration units (**b**) with corresponding codes.

Shannon entropy in the analyzed river catchments (Figure 2a, Table 3) demonstrates clear seasonal variability, reflecting different levels of unpredictability in the distributions of temperature and precipitation throughout the year. The highest entropy values were observed during winter months, particularly in January and February, suggesting increased uncertainty in hydrometeorological conditions during this period.

Entropy values decrease during the spring and summer, reaching their lowest levels in April and May, which indicates a more predictable character of climate variability in these months. The Barycz catchment exhibits one of the lowest April entropy values (3.726), pointing to more stable hydrological conditions in that month. Among the analyzed basins, the highest entropy in January was recorded in the Bóbr River catchment (5.103), indicating high uncertainty and strong fluctuations. The Rega catchment also shows high entropy in January (5.070), possibly linked to significant variability in precipitation and temperature in this region.

The lowest summer entropy values (June–August) were found for the Dziwna and Barycz catchments, suggesting more stable atmospheric conditions in these areas during the summer. During autumn, entropy gradually increases, indicating greater uncertainty during the transitional period between summer and winter. For example, in the Nysa Kłodzka catchment, entropy values in August (4.498) and September (4.463) are higher than in previous summer months, possibly reflecting irregular rainfall following drought periods.

The Prasata River maintains relatively high entropy levels throughout the year, suggesting considerable variability in hydrometeorological conditions. Data also indicate that Shannon entropy in estuarine catchments, such as the Vistula Lagoon and Martwa Wisła, tends to increase towards the end of the year, potentially due to greater atmospheric instability.

The Vistula River catchment from the San to the Wieprz maintains entropy values above 4.5 for most of the year, indicating high unpredictability in the distribution of temperature and precipitation. In contrast, the Wieprz River shows relatively stable entropy values year-round, which may suggest a more uniform distribution of hydrological uncertainty.

Sections of the Oder River exhibit notable regional differences in entropy values, reflecting climatic variability within the river basin. Overall, the highest instability tends to occur during the late winter–early spring and autumn–winter transitions, which aligns with typical atmospheric dynamics in a temperate climate zone.

In conclusion, the analysis of Shannon entropy across second-order river catchments confirms that the dynamics of temperature and precipitation are strongly seasonal, and entropy values serve as an effective indicator for identifying periods of greater or lesser hydrological predictability.

### 10.2. Analysis of Shannon Entropy Values in the Context of Public Administration Activities

Shannon entropy values (Figure 2b, Table 4) reveal varying degrees of uncertainty in the distribution of temperature and precipitation across individual voivodeships (administrative regions) in Poland, which holds key significance for planning adaptive strategies in the context of climate change.

The highest winter entropy values were observed in the Zachodnio-Pomorskie Voivodeship (4.919 in January) and Warmińsko-Mazurskie Voivodeship (4.895 in February), suggesting significant variability in atmospheric conditions in these regions. Voivodeships with high entropy during the summer months—such as Dolnośląskie (4.769 in July) and Śląskie (4.780 in July)—may be particularly vulnerable to sudden hydrological changes, including both droughts and heavy rainfall events leading to floods.

In regions with elevated entropy, local authorities should prioritize water management strategies, including the construction of retention reservoirs and small-scale water retention systems. Low spring entropy values, such as those recorded in Wielkopolskie (3.753 in April) and Kujawsko-Pomorskie (3.786 in April), may indicate more stable climatic conditions, but also signal prolonged dry periods, increasing the risk of agricultural drought. Drought protection programs in these areas should account for irrigation needs in regions characterized by stable yet low springtime entropy.

The greatest climatic variability tends to occur during transitional seasons, such as in Pomorskie in October (4.356), potentially increasing the risk of intense rainfall and flash floods. In such voivodeships, investments in flood early warning systems and enhancement of drainage infrastructure should be prioritized.

Data show that regions with high winter entropy, such as Warmińsko-Mazurskie (4.834 in December) and Zachodnio-Pomorskie (4.568 in December), may be more susceptible to temperature fluctuations and heavy snowfall. Authorities in these regions should focus on modernizing snow removal systems and developing flood control infrastructure in preparation for sudden snowmelt events.

The average summer entropy across most voivodeships ranges between 4.5 and 4.7, indicating relatively stable yet variable weather patterns that require flexible water management strategies. Voivodeships with elevated summer entropy, such as Lubelskie (4.441 in July) and Świętokrzyskie (4.711 in July), may experience irregular precipitation, necessitating the development of stormwater retention infrastructure.

In light of projected increases in entropy over the coming decades due to climate change, public administration should adopt strategies based on long-term forecasting. Local governments are encouraged to integrate entropy data into emergency management systems to better respond to extreme weather events. In regions with high autumn entropy, such as Małopolskie (4.510 in December), enhanced monitoring of river and stream levels is recommended to mitigate the risk of torrential rainfall.

Furthermore, entropy data may serve as a valuable tool for spatial planning, helping identify areas most at risk of floods and droughts. Regions with consistently low entropy throughout the year, such as Opolskie Voivodeship (average entropy ~4.2), may be less prone to abrupt changes, yet still require adaptive measures to build resilience against potential climate-related impacts.

### 10.3. Recommendations for Public Administration in the Context of Drought and Flood Protection and Climate Change Adaptation

Based on the analysis of Shannon entropy values (Table 4), the following recommendations for public administration regarding water resource management and climate change adaptation can be formulated. These recommendations are summarized in Table 5.

## 11. Trend and Seasonal Variability of Shannon Entropy in the Context of Climate Change

**Figure 3:**
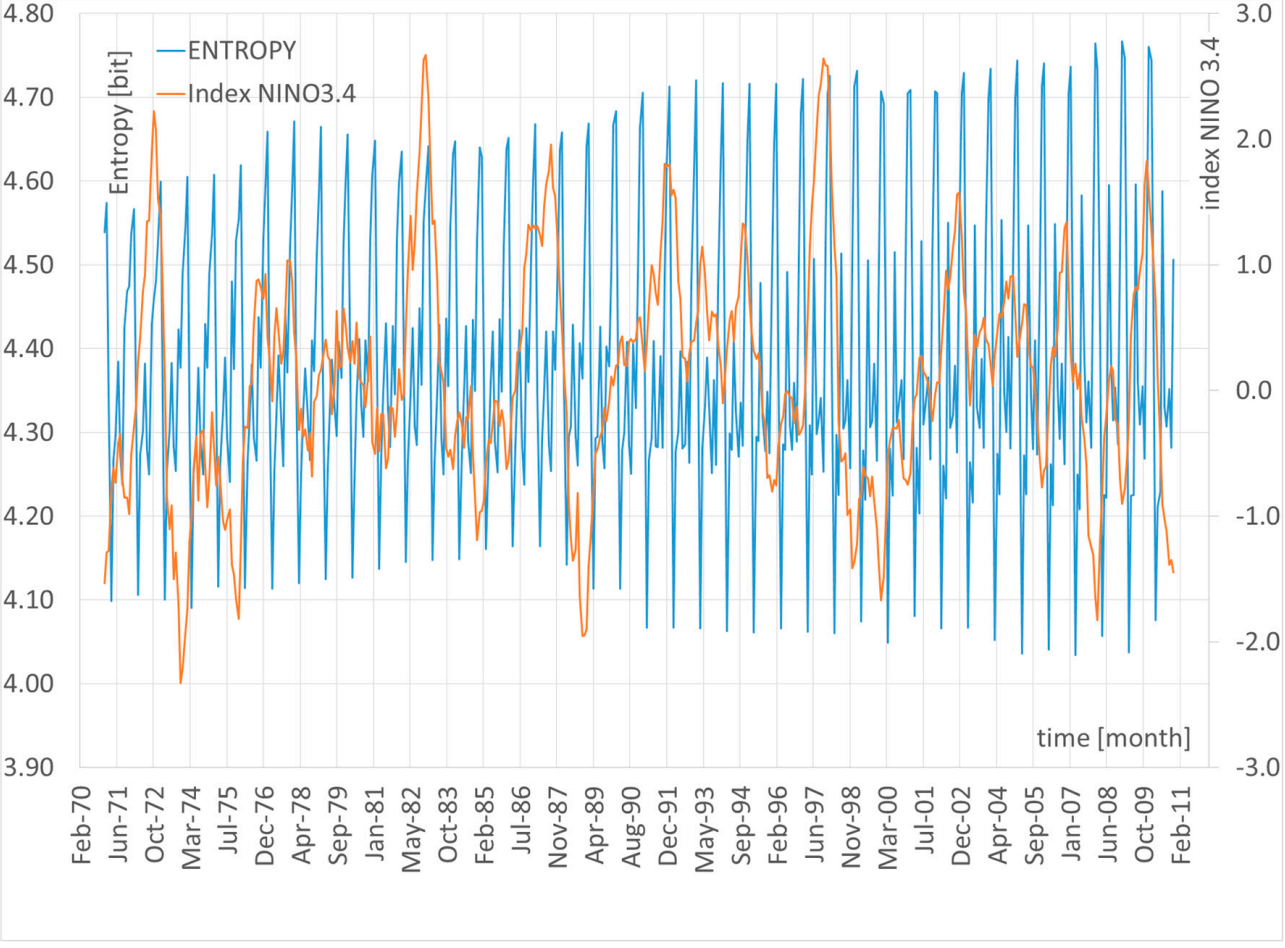
Average Shannon entropy in the years 1971–2010.

Shannon entropy values exhibit a generally increasing trend across most months, indicating growing climate variability over the analyzed period of 1901–2010 (Table 6, Figure 3). The most pronounced increases in entropy were observed during the summer months, particularly in July and August, where values reached their highest levels in recent decades. This suggests increasing instability in the joint distribution of temperature and precipitation.

In winter months (January, February, December), entropy also shows an upward trend, albeit at a more moderate pace, implying that atmospheric variability during winter is rising more slowly than in summer. The lowest entropy values are recorded in the spring months (April and May), which may reflect a more predictable climatic regime during this time of year.

Maximum entropy values observed in February in recent decades (e.g., 4.747 in 2009) may indicate increased instability of winter weather conditions, likely linked to more variable snow precipitation and temperatures. The rise in summer entropy could be attributed to increasingly irregular rainfall patterns and extreme heatwaves, consistent with observed climate change dynamics.

The period from 1940 to 2010 is characterized by a marked increase in entropy in October and November, suggesting greater variability in autumn weather, potentially related to more frequent storms and heavy rainfall. The increase in entropy for summer months—particularly July (e.g., from 4.385 in 1901–1971 to 4.588 in 1940–2010)—confirms a rising irregularity in seasonal precipitation, which may elevate the risk of droughts and flash floods.

Conversely, a slight decline in entropy in May and June in recent decades may point to more stable weather patterns during these months, although this could also result from fewer anomalies occurring in this period. The varying rates of entropy increase across different months indicate that climate change does not affect all seasons equally—variability is most significant during summer and autumn.

In transitional months such as March and September, entropy grows at a moderate rate, suggesting that while changes are observable, these periods remain relatively stable. February shows some of the highest entropy levels across the entire time series, which may point to increasingly erratic snow and temperature conditions, leading to more unpredictable winters.

High entropy values in October and November in recent decades indicate a potential rise in extreme weather events, such as intense autumn storms. The overall upward trend in entropy is consistent with global observations of climate change, confirming increasing instability in weather patterns.

The dynamic behavior of entropy implies that we can expect further increases in weather unpredictability in the future, particularly during the summer and autumn seasons. This underscores the necessity of adjusting water management strategies and strengthening climate adaptation policies.

**Figure 4:**
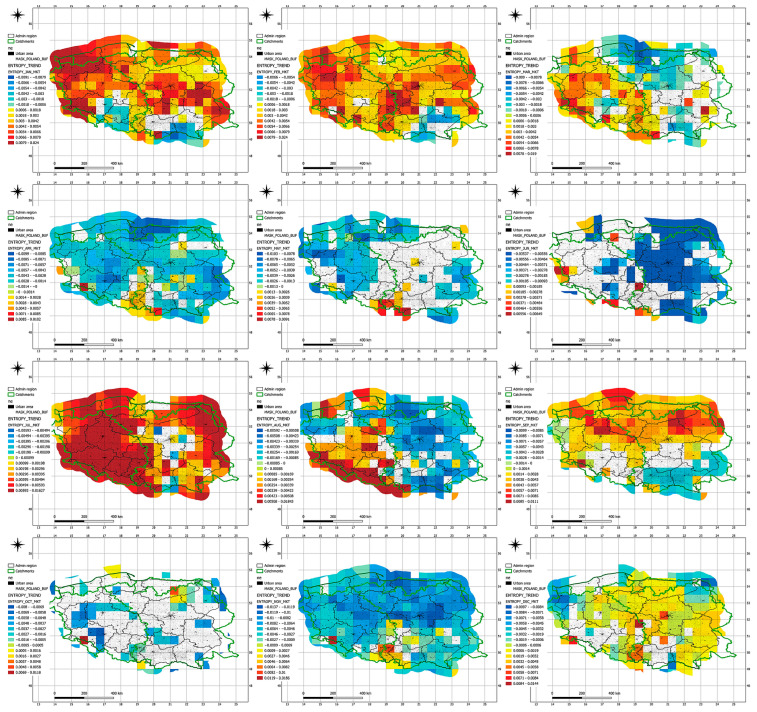
Results of the Mann–Kendall test determined at a 5% significance level identifying seasonal trends in Shannon entropy [bits/season].

Analyzing the Shannon entropy values for the period 1901–2010 allows for the identification of key dynamic indicators (Table 7, Figure 4). By calculating the difference between the first period (1901–1971) and the most recent one (1940–2010), it is possible to estimate the entropy increase over a 40-year span. The greatest rise in entropy occurred in January (+0.221) and July (+0.203), indicating increased unpredictability in winter and summer. The most significant decrease in entropy was recorded in November (–0.188) and October (–0.073), which may suggest greater regularity in atmospheric conditions during autumn. The highest average rate of entropy increase per decade was observed in January (+0.055) and July (+0.051), potentially reflecting growing seasonal variability in response to climate change. Transitional months, such as March, August, and September, show only slight increases in entropy, suggesting that climate variability during these periods is lower compared to winter and summer.

**Figure 5:**
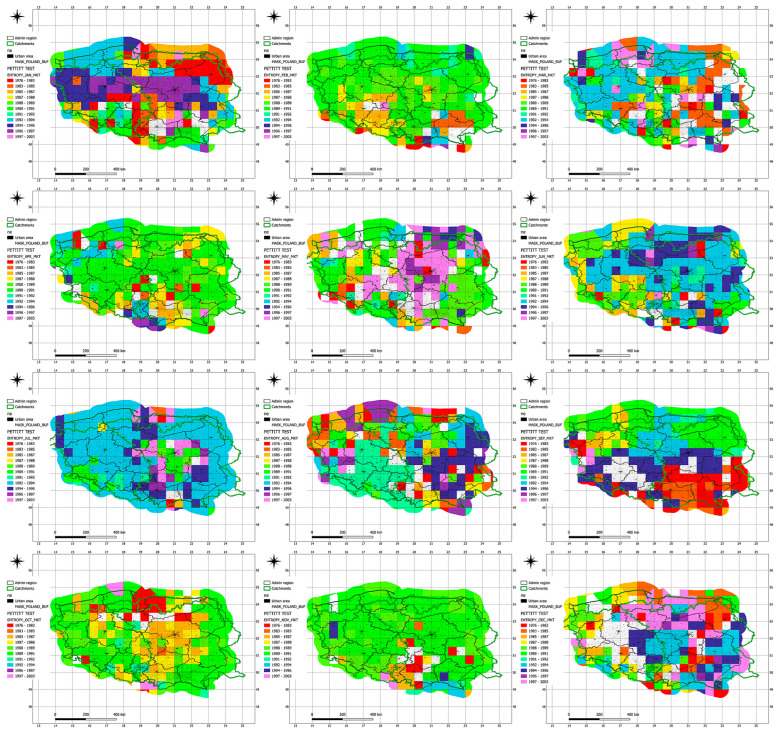
Results of the Pettitt test at a 5% significance level identifying the years in which changes in seasonal trends of Shannon entropy occurred.

The analysis of entropy trend changes across individual voivodeships (Figure 5, Table 8) indicates that the most significant shifts occurred during the 1980s and 1990s, which may reflect the influence of global climate change as well as local environmental transformations. The earliest trend changes were recorded in the Świętokrzyskie Voivodeship (September 1982) and Silesian Voivodeship (January 1983), possibly signaling early signs of climate variability in these regions. The most recent changes occurred in the Kuyavian-Pomeranian (March 1996), Warmian-Masurian (December 1996), and again in Świętokrzyskie (May and July 1996), which may suggest a later adaptation of these areas to new climatic dynamics.

Voivodeships such as Masovian, Pomeranian, and Podlaskie show a wide temporal dispersion of trend change years, indicating a gradual and irregular influence of climate change in these regions. In contrast, voivodeships with relatively clustered changes during similar periods—such as Lower Silesian (1989–1992) and Lubusz (1990–1995)—may share more homogeneous climatic and hydrological conditions affecting entropy.

Frequent trend changes in the period 1989–1992 across multiple voivodeships may correspond with an intensification of weather anomalies in Central Europe during that time, including an increased frequency of droughts and floods. Eastern regions (e.g., Lublin, Podlaskie, Subcarpathian) tend to exhibit earlier trend changes, possibly due to greater sensitivity to continental climatic influences. Western voivodeships (e.g., West Pomeranian, Lubusz, Greater Poland) show changes primarily between 1990 and 1995, suggesting relatively greater climatic stability in those regions until the 1990s.

The clustering of trend changes during 1994–1996 in voivodeships such as Kuyavian-Pomeranian, Warmian-Masurian, and Świętokrzyskie may indicate a delayed response of these areas to emerging climate patterns.

### 11.1. The Relationship Between Climate Variability and Global Climate Change Through the Lens of Shannon Entropy

To gain a deeper understanding of the link between local climate variability in Poland and global climate trends, an additional analysis was conducted to explore the relationship between Shannon entropy values and selected global variables, such as the global land–ocean surface temperature anomaly (NASA Global Mean Estimates—Land–Ocean Temperature Index) and the ENSO index (NINO3.4). For the period 1941–2010, a statistically significant positive Pearson correlation was found between entropy and global temperature rise: ρ = 0.650 (*p* < 0.05) in summer and ρ = 0.826 (*p* < 0.05) in winter, suggesting that intensifying climate change may be contributing to increased weather instability in Poland.

To investigate the potential direction of this relationship, a Granger causality test was applied to assess whether the NINO3.4 index could be considered a predictor of changes in Shannon entropy. The analysis was conducted over a broad range of time lags, from 1 to 24 months. For lags of 8, 9, and 11 months, the test indicated statistical significance (e.g., *p* = 0.0453 for a 9-month lag); however, for most other lags, the results were not significant, and the F-values did not exceed the critical threshold at the 0.05 significance level.

The fact that significance emerged only for selected lags may indicate temporal instability in the relationship or the presence of nonlinear and seasonal mechanisms linking global ENSO variability with local climate entropy. These results do not provide strong evidence of Granger-type causality but may point to lagged, seasonally dependent teleconnection effects. Notably, periods of intense El Niño events (e.g., 1982–1983, 1997–1998) coincided with marked increases in entropy observed in Poland.

### 11.2. Attractor of the Mean Shannon Entropy

To describe the dynamics of Shannon entropy variability, a three-dimensional phase space analysis was performed using the coordinates [X(t),Y(t+τ),Z(t+2τ)]. The foundation for constructing the phase space plot was the set of average entropy values calculated for moving 40-year periods, separately for each of the 12 months. These values enabled the reconstruction of the system’s trajectory in state space through the application of the time-delay embedding method [91,92,93]. The time delay τ was determined based on the analysis of the autocorrelation function of entropy, selecting its first minimum as the optimal value (τ = 3 months), which indicates the lowest level of information redundancy. The calculated τ represents the optimal temporal lag at which the dependency between variables is strongest, thus allowing for the reconstruction of the system’s hidden dynamic structure.

**Figure 6:**
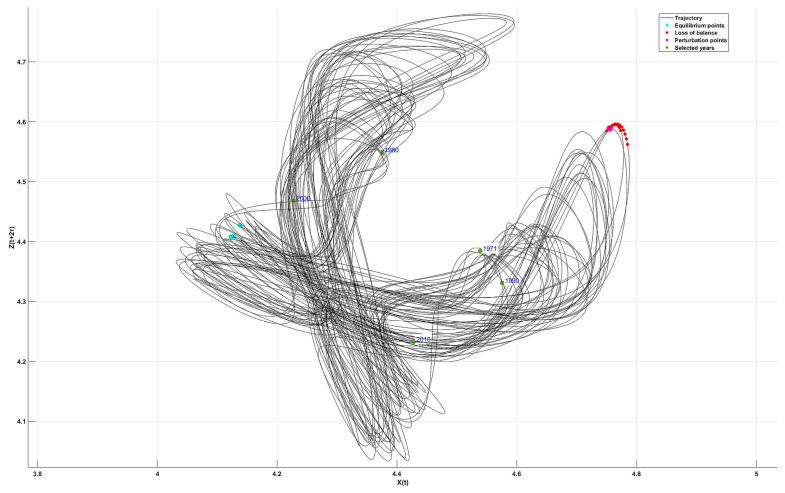
Attractor of the mean Shannon entropy.

The attractor (Figure 6) illustrates the trajectory of entropy evolution over time, highlighting regions of stable and unstable states within the system. Interpreting the attractor allows for the identification of equilibrium points (stable states), transitional points (metastable states), and perturbation points (extreme changes) [47,94]. Transitional points represent system states in which small disturbances may trigger a shift to a different dynamic regime, while perturbation points indicate moments of maximum instability, potentially associated with extreme climatic events.

Equilibrium points (Table 9), with X(t), Y(t+τ), and Z(t+2τ) values oscillating around 4.124–4.138, 4.387–4.431, and 4.408–4.428, respectively, reflect relatively stable climatic conditions during certain time intervals. Transitional points (X = 4.768, Y = 4.053, Z = 4.588) mark regions where entropy undergoes irregular fluctuations, possibly indicating the influence of climate anomalies. Perturbation points (X = 4.755, Y = 4.056, Z = 4.589) signal the moments of the greatest fluctuations, where the system may transition to a new dynamic state—potentially linked to shifts in global climate trends.

For selected years, a gradual shift in entropy values is observable, suggesting a long-term trend toward increasing instability of the climate system. For instance, in 1971, the coordinates (X = 4.539, Y = 4.099, Z = 4.3845) were closer to the equilibrium zone, indicating more predictable conditions. In subsequent decades (1980, 1990), entropy values progressively move toward greater instability, potentially reflecting intensifying weather anomalies. By 2000 (X = 4.707, Y = 4.049, Z = 4.515) and 2010 (X = 4.760, Y = 4.076, Z = 4.588), the system approaches values characteristic of transitional and perturbation points, suggesting a growing irregularity in the distribution of temperature and precipitation.

The observed changes may indicate a long-term trend toward increased climatic chaos, reinforcing the hypothesis of intensifying extreme weather events. The attractor illustrates the dynamic structure of the system, revealing both periodic patterns and transitions to states of greater instability. Phase–space trajectory analysis can assist in forecasting future changes in climate variability by identifying transitional periods and potential tipping points. The characteristic values of the attractor suggest that the system exhibits features of chaotic behavior, where even small variations in initial conditions can lead to significant differences in system dynamics.

The data indicate that the climate is shifting from a zone of relative stability toward a state of heightened unpredictability. The increase in entropy in the years 2000 and 2010, compared to earlier periods, may signal a transition to a new climatic regime characterized by greater instability. In summary, the attractor constructed from the phase–space diagram and average entropy values highlights key transitional moments in climate dynamics, and its analysis may serve as a valuable tool for anticipating future climatic shifts.

## 12. Summary

This study presents an innovative approach to long-term climate variability analysis in Poland by using Shannon entropy as an integrated indicator of climate uncertainty. A key feature of this methodology is the application of a joint distribution of temperature and precipitation, constructed using the Clayton copula function. The marginal distributions—normal for temperature and gamma for precipitation—are combined in a way that captures asymmetric dependencies in the lower tails, which is particularly important for analyzing extreme events such as droughts.

The use of the bootstrap technique and numerical integration over a 0.25° × 0.25° spatial grid enabled precise estimation of Shannon entropy in a bivariate setting. This allowed for a high-resolution assessment of spatial and seasonal climate variability. Another novel element of the analysis was the reconstruction of entropy trajectories in phase space using the time-delay embedding method (τ = 3 months), which made it possible to identify equilibrium, transitional, and perturbation points—critical to understanding dynamic shifts in the climate system.

The results indicate a systematic increase in Shannon entropy over the study period (1901–2010), especially during the winter and summer months. The greatest increases were observed in January (+0.221 bits) and July (+0.203 bits), reflecting growing unpredictability in both winter and summer atmospheric conditions. The rate of entropy growth in these months reached +0.055 and +0.051 bits per decade, respectively, highlighting a clear intensification of variability. In contrast, spring months such as April and May showed slight decreases in entropy (e.g., −0.056 bits in May), which may indicate periods of relative climate stabilization.

A crucial part of the analysis involved assessing the relationship between entropy and global climate indicators. A strong positive correlation was found between Shannon entropy and the NASA GISTEMP Land–Ocean Temperature Index: ρ = 0.826 in winter and ρ = 0.650 in summer (both *p* < 0.05). This suggests that global temperature rise contributes to increased local climate instability. For the ENSO index (NINO3.4), the Granger causality test for lags of 1–24 months showed statistical significance only in isolated cases (e.g., *p* = 0.0453 at a 9-month lag), indicating a lack of consistent causality—suggesting co-variability rather than a directional relationship.

Phase space analysis revealed entropy trajectories passing through equilibrium states (X ≈ 4.124–4.138), unstable points (X = 4.768), and perturbation points (X = 4.755). The temporal shift in these values—especially in 2000 and 2010—signals a transition of the climate system into a more unstable regime, characteristic of chaotic systems. This dynamic supports the hypothesis that Poland’s climate is evolving from a more predictable regime into an increasingly irregular and complex one.

The methodology employed—combining statistics, information theory, and nonlinear dynamic systems analysis—offers a new tool for monitoring climate change. Entropy values can be used as indicators of hydrological risk and serve as a basis for early warning systems. These findings are particularly useful for public administration, climate adaptation planning, and water resource management. The increase in entropy signals a growing risk of extreme weather events such as droughts, flash floods, and heatwaves.

Although this study has high applied value, certain limitations must be acknowledged. The 0.25° × 0.25° resolution may not fully capture local-scale phenomena, and the choice of copula and marginal distributions affects sensitivity. Furthermore, the model does not account for anthropogenic variables such as land use changes or urbanization.

In conclusion, Shannon entropy—derived from the joint distribution of temperature and precipitation—proves to be an effective tool for diagnosing climate instability. This study demonstrates that Poland’s climate is becoming increasingly unpredictable, as confirmed by both historical data and its correlation with global trends. The results hold significant potential for broader applications in climate monitoring, spatial adaptation, and crisis management in response to climate change.

## Figures and Tables

**Table 1:** The applied Archimedean copula function and dependence measure.

Bivariate Copula Function	
maxu−θ+v−θ−1−1θ , where: θ∈[−1,∞)\{0}	(4)
Kendall’s τ	
$\frac{\theta }{\theta +2}$	(5)

**Table 2:** Summary of the average values of the parameter θ.

Month	JAN	FEB	MAR	APR	MAY	JUN	JUL	AUG	SEP	OCT	NOV	DEC
θ	0.361	0.180	0.091	0.045	0.041	0.033	0.025	0.037	0.016	0.066	0.295	0.293

**Table 3:** Mean values of Shannon Entropy calculated from the joint distribution of precipitation and temperature for second-order river basins for the years 1941–2010.

CODE	NAME	JAN	FEB	MAR	APR	MAY	JUN	JUL	AUG	SEP	OCT	NOV	DEC
24	Nysa Kłodzka	4.620	4.663	4.389	3.996	4.334	4.286	4.730	4.498	4.463	4.384	4.183	4.249
28	Barycz	4.664	4.641	4.368	3.726	4.043	3.986	4.628	4.107	4.054	4.179	4.039	4.461
35	Cieśnina Dziwna	4.812	4.785	4.319	3.925	3.956	3.822	4.395	4.045	4.083	4.144	4.078	4.370
44	Parsęta	4.970	4.916	4.590	4.077	4.033	4.031	4.643	4.285	4.550	4.411	4.347	4.745
45	Odra od Baryczy do Bobru (l)	4.769	4.780	4.395	3.898	4.130	4.008	4.722	4.199	4.166	4.166	4.095	4.485
45	Przymorze od Parsęty do Wieprzy	4.900	4.859	4.434	4.069	4.488	4.277	4.729	4.543	4.466	4.564	4.405	4.607
46	Wieprza	4.879	4.823	4.450	3.979	4.140	4.052	4.562	4.332	4.437	4.468	4.359	4.690
51	Odra od Bobru do Warty (p)	4.817	4.813	4.375	4.059	4.181	4.061	4.674	4.208	4.122	4.176	4.084	4.472
51	Zalew Wiślany do Nogatu	4.787	4.917	4.296	3.886	4.171	4.128	4.464	4.357	4.421	4.411	4.319	4.881
52	Nogat	4.911	4.769	4.247	3.809	4.078	4.094	4.392	4.191	4.417	4.132	4.175	4.687
55	Zalew Wiślany od Elbląga do Pasłęki	4.903	4.959	4.369	3.851	4.155	4.102	4.455	4.336	4.467	4.480	4.392	4.949
62	Świsłocz (l)	4.681	4.613	4.357	4.018	4.130	4.159	4.634	4.304	4.137	4.237	4.118	4.355
64	Bóbr	5.103	5.012	4.620	4.293	4.397	4.205	4.853	4.443	4.461	4.533	4.365	4.682
64	Czarna Hańcza (l)	4.711	4.638	4.373	3.980	4.076	4.188	4.733	4.425	4.178	4.351	4.230	4.345
72	Lechnawa	4.551	4.643	4.606	4.189	4.286	4.290	4.442	4.243	4.479	4.534	4.404	4.756
77	Odra do Nysy Kłodzkiej (l)	4.504	4.624	4.307	4.013	4.285	4.193	4.619	4.204	4.328	4.343	4.189	4.340
84	Rega	5.070	4.962	4.606	4.137	4.112	3.962	4.567	4.331	4.386	4.433	4.364	4.667
91	Odra od Nysy Kłodzkiej do Baryczy (p)	4.602	4.642	4.280	3.899	4.227	4.130	4.704	4.272	4.196	4.260	4.092	4.340
92	Wisła od Sanu do Wieprza (p)	4.666	4.731	4.544	4.152	4.099	4.323	4.588	4.298	4.220	4.278	4.335	4.466
96	Orlica (Dzika Orlica)	4.605	4.600	4.257	4.173	4.330	4.159	4.702	4.447	4.311	4.302	4.089	4.362
96	Martwa Wisła	4.683	4.608	4.046	3.925	4.121	3.984	4.433	4.401	4.333	4.340	4.176	4.592
112	Drwęca	4.780	4.876	4.311	3.831	4.108	4.228	4.579	4.176	4.401	4.243	4.188	4.760
112	Pasłęka	5.015	5.019	4.546	3.944	4.175	4.256	4.493	4.388	4.549	4.491	4.387	5.022
114	Odra od Warty do ujścia	4.842	4.837	4.254	3.963	4.017	4.031	4.460	4.032	4.035	4.142	4.011	4.337
144	Wieprz	4.657	4.669	4.560	4.027	4.021	4.187	4.427	4.175	4.135	4.236	4.265	4.460
174	Wisła od Drwęcy do ujścia	4.850	4.773	4.356	3.895	4.085	4.125	4.609	4.157	4.341	4.250	4.226	4.698
175	Wisła od Wieprza do Narwi (p)	4.686	4.655	4.482	3.942	4.127	4.192	4.566	4.097	4.117	4.127	4.260	4.461
188	Przymorze od Wieprzy do Martwej Wisły	4.865	4.815	4.344	3.976	4.162	3.995	4.576	4.400	4.398	4.454	4.382	4.692
198	San	4.552	4.649	4.619	4.173	4.270	4.284	4.468	4.156	4.354	4.387	4.317	4.578
243	Wisła od Narwi do Drwęcy (l)	4.641	4.694	4.224	3.890	4.160	4.247	4.641	4.116	4.174	4.145	4.259	4.593
348	Pregoła	4.900	4.845	4.521	3.907	4.057	4.214	4.576	4.418	4.429	4.388	4.365	4.711
357	Wisła do Sanu	4.776	4.841	4.731	4.281	4.363	4.389	4.682	4.274	4.491	4.460	4.467	4.524
486	Warta	4.687	4.743	4.374	3.849	4.142	4.172	4.665	4.127	4.207	4.237	4.171	4.527
1014	Narew	4.705	4.707	4.389	3.925	4.088	4.196	4.498	4.229	4.186	4.232	4.194	4.462

**Table 4:** Average Shannon entropy values calculated from the joint distribution of precipitation and temperature for voivodeships for the years 1941–2010.

CODE	NAME_	JAN	FEB	MAR	APR	MAY	JUN	JUL	AUG	SEP	OCT	NOV	DEC
2	Dolnośląskie	4.781	4.782	4.418	4.073	4.317	4.192	4.769	4.372	4.318	4.373	4.213	4.461
4	Kujawsko-Pomorskie	4.683	4.746	4.231	3.786	4.079	4.228	4.680	4.168	4.237	4.130	4.147	4.618
6	Lubelskie	4.660	4.669	4.576	4.040	4.026	4.197	4.441	4.160	4.147	4.242	4.271	4.471
8	Lubuskie	4.816	4.825	4.404	3.976	4.155	4.065	4.694	4.182	4.151	4.180	4.113	4.512
10	Łódzkie	4.609	4.680	4.350	3.904	4.252	4.225	4.670	4.086	4.162	4.216	4.310	4.484
12	Małopolskie	4.733	4.808	4.692	4.286	4.366	4.383	4.647	4.277	4.492	4.428	4.400	4.510
14	Mazowieckie	4.689	4.671	4.325	3.921	4.066	4.205	4.503	4.190	4.136	4.150	4.227	4.510
16	Opolskie	4.499	4.609	4.257	3.896	4.213	4.120	4.593	4.117	4.293	4.297	4.071	4.365
18	Podkarpackie	4.605	4.672	4.669	4.172	4.292	4.314	4.494	4.191	4.383	4.391	4.334	4.612
20	Podlaskie	4.685	4.674	4.374	3.910	4.081	4.168	4.579	4.305	4.167	4.284	4.173	4.359
22	Pomorskie	4.840	4.774	4.293	3.926	4.127	4.039	4.524	4.301	4.384	4.356	4.276	4.695
24	Śląskie	4.689	4.807	4.561	4.185	4.360	4.324	4.780	4.226	4.386	4.450	4.449	4.484
26	Swiętokrzyskie	4.745	4.780	4.610	4.106	4.231	4.321	4.711	4.128	4.366	4.304	4.344	4.449
28	Warmińsko-Mazurskie	4.880	4.895	4.503	3.896	4.095	4.218	4.504	4.338	4.469	4.386	4.333	4.834
30	Wielkopolskie	4.660	4.697	4.333	3.753	4.111	4.135	4.633	4.116	4.175	4.217	4.098	4.524
32	Zachodniopomorskie	4.919	4.878	4.456	4.022	4.073	4.014	4.571	4.186	4.294	4.316	4.234	4.568

**Table 5:** Recommendations for public administration in water resource management and climate change adaptation.

Regional Water Management Strategies	
Provinces with High Winter Entropy (e.g., Zachodniopomorskie, Warmińsko-Mazurskie).	Expansion of retention systems and flood-control reservoirs to mitigate the impact of sudden snowmelt and rainfall. Modernization of flood embankments and drainage infrastructure in areas most at risk of inundation. Implementation of smart water management systems to monitor river levels and forecast flood risks.
Provinces with High Summer Entropy (e.g., Dolnośląskie, śląskie, Swiętokrzyskie, Lubelskie).	Development of small-scale water retention programs, including the construction of ponds and reservoirs to store water during drought periods. Grants for rainwater harvesting systems for households and businesses. Support for agriculture through drip irrigation systems and water-saving technologies.
Provinces with Low Spring Entropy (e.g., Kujawsko-Pomorskie, Wielkopolskie).	Monitoring of prolonged dry spells and implementation of field irrigation systems. Development of local-level water management plans, adapted to specific soil and climatic conditions. Combating soil degradation by expanding green spaces and protecting forested areas.
Development of Monitoring and Forecasting Systems	
Modern Early Warning Systems for Extreme Weather Events.	Installation of precipitation and water level sensors in regions with high climatic variability. Deployment of AI-based forecasting systems capable of predicting heavy rainfall and drought periods. Improvement of hydrological models by incorporating Shannon entropy data for enhanced emergency response planning.
Spatial and Urban Adaptation	
Climate-Resilient Urban Planning.	Limiting urban sealing through green roofs and permeable surfaces. Expansion of urban green spaces to reduce the urban heat island effect and improve stormwater infiltration. Development of flood risk maps based on entropy analysis to guide infrastructure planning.
Education and Public Engagement	
Raising Public Awareness of Climate Change Impacts.	Educational campaigns on water conservation and efficiency. Subsidy programs for households to install rainwater storage and management systems. Promotion of sustainable agricultural practices in drought-prone areas.
Interregional Cooperation and Administrative Integration	
Coordination Between National and Local Authorities.	Establishment of regional crisis management centers to analyze entropy and climatic variability data. Inter-voivodeship cooperation among regions with similar climate conditions, e.g., joint investments in water systems. Integration of water policy with regional development strategies, ensuring alignment of investments with projected climate scenarios.

**Table 6:** Mean monthly Shannon entropy values calculated for 70-year periods.

Period	JAN	FEB	MAR	APR	MAY	JUN	JUL	AUG	SEP	OCT	NOV	DEC
1901–1971	4.539	4.574	4.342	4.099	4.267	4.297	4.385	4.272	4.237	4.425	4.469	4.474
1902–1972	4.537	4.566	4.335	4.106	4.274	4.301	4.382	4.280	4.249	4.430	4.459	4.480
1903–1973	4.528	4.599	4.333	4.100	4.253	4.303	4.383	4.282	4.254	4.423	4.377	4.489
1904–1974	4.543	4.605	4.326	4.090	4.250	4.294	4.377	4.292	4.250	4.429	4.377	4.490
1905–1975	4.536	4.607	4.353	4.116	4.264	4.324	4.389	4.293	4.241	4.480	4.376	4.529
1906–1976	4.555	4.619	4.354	4.114	4.267	4.319	4.382	4.294	4.266	4.438	4.377	4.532
1907–1977	4.594	4.659	4.346	4.114	4.253	4.320	4.392	4.316	4.260	4.447	4.371	4.523
1908–1978	4.590	4.671	4.359	4.120	4.250	4.322	4.376	4.317	4.267	4.410	4.373	4.514
1909–1979	4.583	4.665	4.359	4.125	4.258	4.327	4.387	4.329	4.296	4.408	4.365	4.524
1910–1980	4.598	4.656	4.369	4.127	4.244	4.340	4.412	4.331	4.295	4.410	4.361	4.530
1911–1981	4.607	4.648	4.372	4.137	4.269	4.348	4.430	4.327	4.283	4.427	4.346	4.530
1912–1982	4.598	4.636	4.395	4.145	4.266	4.347	4.425	4.307	4.285	4.448	4.357	4.550
1913–1983	4.592	4.642	4.389	4.148	4.263	4.350	4.429	4.293	4.250	4.436	4.355	4.544
1914–1984	4.633	4.648	4.388	4.149	4.276	4.348	4.427	4.289	4.252	4.435	4.350	4.529
1915–1985	4.640	4.629	4.368	4.161	4.278	4.361	4.420	4.290	4.252	4.435	4.349	4.521
1916–1986	4.639	4.652	4.346	4.164	4.283	4.358	4.422	4.283	4.238	4.425	4.360	4.509
1917–1987	4.603	4.668	4.347	4.164	4.292	4.349	4.420	4.285	4.254	4.421	4.375	4.498
1918–1988	4.637	4.658	4.353	4.142	4.295	4.307	4.428	4.295	4.260	4.406	4.364	4.502
1919–1989	4.642	4.669	4.357	4.114	4.293	4.295	4.426	4.289	4.257	4.402	4.379	4.489
1920–1990	4.667	4.683	4.368	4.113	4.280	4.299	4.408	4.285	4.250	4.406	4.329	4.490
1921–1991	4.665	4.706	4.381	4.067	4.267	4.293	4.409	4.283	4.283	4.391	4.282	4.485
1922–1992	4.621	4.713	4.366	4.067	4.277	4.301	4.397	4.281	4.286	4.385	4.263	4.479
1923–1993	4.614	4.720	4.377	4.066	4.281	4.333	4.389	4.330	4.252	4.362	4.261	4.469
1924–1994	4.625	4.717	4.363	4.063	4.299	4.279	4.408	4.325	4.271	4.336	4.284	4.489
1925–1995	4.649	4.716	4.391	4.061	4.295	4.290	4.479	4.319	4.277	4.349	4.275	4.500
1926–1996	4.646	4.717	4.393	4.066	4.286	4.279	4.491	4.308	4.279	4.359	4.289	4.508
1927–1997	4.682	4.722	4.402	4.062	4.308	4.250	4.507	4.298	4.315	4.341	4.253	4.521
1928–1998	4.705	4.726	4.389	4.061	4.297	4.226	4.514	4.305	4.314	4.363	4.257	4.500
1929–1999	4.713	4.732	4.402	4.074	4.278	4.220	4.506	4.307	4.313	4.382	4.266	4.505
1930–2000	4.707	4.692	4.398	4.049	4.281	4.225	4.515	4.312	4.334	4.362	4.268	4.508
1931–2001	4.704	4.709	4.427	4.081	4.282	4.203	4.529	4.310	4.333	4.366	4.268	4.515
1932–2002	4.708	4.705	4.422	4.066	4.260	4.222	4.550	4.305	4.320	4.380	4.276	4.525
1933–2003	4.706	4.729	4.414	4.067	4.265	4.216	4.547	4.330	4.306	4.388	4.282	4.529
1934–2004	4.699	4.735	4.413	4.052	4.275	4.226	4.554	4.347	4.300	4.414	4.281	4.506
1935–2005	4.698	4.744	4.414	4.036	4.273	4.226	4.547	4.344	4.280	4.410	4.274	4.482
1936–2006	4.714	4.741	4.413	4.041	4.262	4.213	4.548	4.352	4.293	4.382	4.262	4.498
1937–2007	4.705	4.737	4.425	4.034	4.250	4.208	4.583	4.386	4.312	4.361	4.281	4.522
1938–2008	4.764	4.733	4.421	4.057	4.225	4.222	4.596	4.383	4.314	4.353	4.286	4.515
1939–2009	4.767	4.747	4.430	4.038	4.225	4.225	4.597	4.362	4.309	4.355	4.269	4.512
1940–2010	4.760	4.744	4.425	4.076	4.211	4.229	4.588	4.331	4.307	4.352	4.281	4.506

**Table 7:** Changes in the average monthly Shannon entropy calculated for 70-year periods.

Month	Entropy Change (1940–2010)—(1901–1971)	Average Growth Rate (per Decade)
	[bits]	[bits/10 years]
JAN	+0.221 (4.760–4.539)	0.055
FEB	+0.170 (4.744–4.574)	0.043
MAR	+0.083 (4.425–4.342)	0.021
APR	−0.023 (4.076–4.099)	−0.006
MAY	−0.056 (4.211–4.267)	−0.014
JUN	−0.068 (4.229–4.297)	−0.017
JUL	+0.203 (4.588–4.385)	0.051
AUG	+0.059 (4.331–4.272)	0.015
SEP	+0.070 (4.307–4.237)	0.018
OCT	−0.073 (4.352–4.425)	−0.018
NOV	−0.188 (4.281–4.469)	−0.047
DEC	+0.032 (4.506–4.474)	0.008

**Table 8:** Average years of change in the monthly trend of Shannon entropy.

CODE	Name_	JAN	FEB	MAR	APR	MAY	JUN	JUL	AUG	SEP	OCT	NOV	DEC
2	Dolnośląskie	1988	1987	1991	1990	1990	1989	1992	1994	1991	1989	1989	1989
4	Kujawsko-Pomorskie	1994	1989	1990	1990	1993	1996	1994	1988	1992	1984	1990	1996
6	Lubelskie	1992	1989	1991	1989	1990	1994	1996	1991	1988	1989	1989	1994
8	Lubuskie	1995	1990	1993	1992	1990	1992	1993	1994	1991	1990	1991	1990
10	Łódzkie	1989	1991	1991	1988	1997	1993	1996	1989	1993	1988	1988	1994
12	Małopolskie	1992	1989	1985	1989	1991	1991	1992	1990	1984	1988	1987	1989
14	Mazowieckie	1995	1989	1988	1990	1995	1993	1994	1992	1994	1987	1990	1996
16	Opolskie	1992	1987	1990	1988	1990	1989	1993	1992	1989	1990	1989	1985
18	Podkarpackie	1991	1987	1990	1992	1991	1991	1994	1992	1985	1990	1991	1995
20	Podlaskie	1984	1991	1986	1989	1994	1992	1994	1994	1990	1988	1990	1989
22	Pomorskie	1991	1990	1996	1991	1991	1992	1995	1992	1990	1987	1990	1993
24	Śląskie	1983	1989	1991	1990	1988	1995	1994	1991	1991	1990	1989	1991
26	Świętokrzyskie	1990	1991	1987	1987	1996	1987	1996	1994	1982	1988	1983	1989
28	Warmińsko-Mazurskie	1988	1991	1992	1989	1995	1994	1994	1989	1992	1985	1990	1996
30	Wielkopolskie	1992	1990	1993	1990	1992	1992	1994	1991	1992	1989	1990	1991
32	Zachodniopomorskie	1995	1990	1993	1990	1995	1989	1994	1989	1987	1988	1989	1986

**Table 9:** Coordinate values of the characteristic points of the attractor.

No.	Continent	X(t)	Y(t + τ)	Z(t + 2τ)
1	Equilibrium Points	4.124	4.387	4.408
		4.127	4.413	4.410
		4.138	4.431	4.428
3	Unstable Points	4.768	4.053	4.588
4	Perturbation Points	4.755	4.056	4.589
Selected Years				
5	1971	4.539	4.099	4.3845
6	1980	4.598	4.127	4.412
7	1990	4.667	4.113	4.408
8	2000	4.707	4.049	4.515
9	2010	4.760	4.076	4.588

## Data Availability

Dataset available on request from the author.
